# The Performance of Ultrafiltration Process to Further Refine Lactic Acid from the Pre-Microfiltered Broth of Kitchen Waste Fermentation

**DOI:** 10.3390/membranes13030330

**Published:** 2023-03-13

**Authors:** Yan Guo, Chenglong Li, Hongjun Zhao, Xiaona Wang, Ming Gao, Xiaohong Sun, Qunhui Wang

**Affiliations:** 1 Department of Environmental Engineering, University of Science and Technology Beijing, 30 Xueyuan Road, Haidian District, Beijing 100083, China; 2 Beijing Agro-Biotechnology Research Center, Beijing Academy of Agriculture and Forestry Sciences, Beijing 100097, China

**Keywords:** kitchen waste, fermentation broth, lactic acid, ultrafiltration, refinement, flushing

## Abstract

Lactic acid (LA) is an important chemical material facing rapid demand in recent years. The oriented fermentation of kitchen waste is a promising route for economic LA production. However, the refinement of LA from fermentation broth is a spiny issue. In this study, the performance of ultrafiltration (UF) process for the refinement of LA from the pre-microfiltered broth of kitchen waste fermentation was first investigated. The results showed that with 50 KDa polyethersulfone membrane, under the optimum pressure of 120 KPa, the pH of 6.0, and the backflushing mode with the deionized water for 3 min, the best performance was achieved with the chroma removal efficiency, turbidity removal efficiency, protein removal efficiency and total sugar removal efficiency of 54.3%, 89.8%, 71.7% and 58.5%, respectively, and LA recovery efficiency was 93.6%. The results indicated that the UF process could further effectively refine the pre-microfiltered broth of kitchen waste fermentation, and the combination of microfiltration and UF process is ideal for achieving desirable LA refinement performance. This study verified the feasibility of UF process in LA refinement from pre-microfiltered broth of kitchen waste fermentation, and based on the results, the further exploration of proper post-process to treat UF filtrate for obtaining LA product with higher quality should be explored in the future.

## 1. Introduction

Lactic acid (LA) is one of the organic acids. The chemical formula of the LA is C_3_H_6_O_3_. It has two optical isomers, Levo and Dextro, making itself a chiral molecule. L-isomers are commonly present among living organisms. The extended formula of LA is CH_3_CH(OH)CO_2_H, and it has a molar mass of 90.08 g/mol. Currently, as one of the important chemical materials for industrial applications, such as those related to the food, tanning, petrochemicals, pharmaceuticals, cosmetics and chemicals industry, LA is obtained mainly relying on chemical conversion from petrochemical raw material [1]. With the world’s most urgent mission-carbon neutrality by the middle of the century, as well as the raised awareness of environmental effects in terms of pollution and greenhouse effect, the alternative route for LA production is expected [2,3]. Fortunately, it was reported that LA can be obtained through low-cost fermentation/anaerobic digestion process with the low-cost biomass [4,5]. However, the refinement and consequent concentration of LA from fermented broths are challenging due to the relatively low concentration of LA and complex impurities in the broths [6]. Thus, low cost and concise refinement technologies are urgently needed [7].

Research progress in the LA refinement was remarkable recently, together with the detection methods [8,9], and refinement techniques [6,10]. Various methods for refining LA from fermented broths were explored, including electrodialysis [11], chemical precipitation [12], ion-exchange [13], solvent extraction [14], distillation [15,16], adsorption [17] and membrane processes [6,18]. Especially, the membrane-based technology, characterized as being potentially efficient, cost-effective and eco-friendly, is a promising process [19].

Until now, the refinement of LA existing in the fermented broth was successfully conducted by the nanofiltration (NF) and reverse osmosis (RO) processes [20,21]. However, the pre-treatment of fermented broth is requested due to the high turbidity of broth, which cannot be directly fed to the spiral-wound membrane modules of the NF and RO processes. Commonly, the microfiltration (MF) and ultrafiltration (UF) processes, deemed as the best pre-treatment strategies for removing most components (such as particles, colloids, bacteria and high molecular mass organic matter) causing the membranes fouling [21], can effectively achieve this goal [20,22,23].

Food waste (FW), the main composition of municipal solid waste (MSW), accounts for 37–62% of MSW with a rapid increase trend in China and approximately 12–30% in some developed countries such as USA and UK [24]. Thus, FW is a promising source for energy and resource recovery, due to its high organic content [25]. In recent years, fermentation technology was adopted to recycle the FW through the production of many high-value products such as LA and alcohols existing in the fermented broths [26]. It seemed that the membrane process could be utilized to refine the fermented broth to obtain the high-value-added LA product. However, a concern was that the impact of fermented broths on membrane fouling is several times larger compared to that of the surface water and seawater [27].

In the author’s previous lab-scale research, the MF filtration of the kitchen waste (KW) fermentation broth was conducted. After MF filtration, most of the suspended particles and bacteria in the broth were effectively removed. However, there are still many soluble macromolecular organic impurities in the MF permeate, such as protein substances and residual total sugars. Among them, due to the Maillard reaction, substances such as sugar and protein will produce brown or even black substances, which seriously affects the quality of LA products [28]. UF is a purification process that separates particulate matter from soluble compounds using an ultrafine membrane media. Ultrafiltration is an excellent separation technology for desalination pretreatment, reverse osmosis pretreatment, and wastewater reclamation, as well as for producing potable water. Like MF, UF is based on size exclusion or particle capture. UF can purify and concentrate macromolecular (10^3^–10^6^ Da) solutions, especially protein solutions. Therefore, it is desirable to use UF process to further refine the MF permeates to improve the LA purity.

Thus, in this study, the refinement of pre-microfiltered broth of kitchen waste fermentation was explored through the UF process. Firstly, the refinement performance of four different UF membranes on the simulated broth was compared for selecting the proper UF membrane type. Then, the selected UF membrane was adopted to refine the actual pre-microfiltered broth, and the effect of different operating pressures (0.08 MPa, 0.10 MPa, 0.12 MPa, 0.14 MPa) and different pH (5, 6, 7, 8) on refinement performance were investigated to optimize the operation parameters. Finally, the recovery efficiency of pure water flux of UF membrane under different cleaning modes, cleaning agents and cleaning time was conducted to obtain the optimal scheme for UF membrane cleaning. In addition, the 10 filtration-cleaning cycles were continuously carried out to investigate the effect of cleaning times on the treatment performance of UF membrane. Under each condition, the chroma removal efficiency (CRE), the turbidity removal efficiency (TRE), total sugar removal efficiency (SRE), protein removal efficiency (PRE) and LA recovery efficiency (LRE) were investigated.

## 2. Materials and Methods

### 2.1. Fermented Broth

#### 2.1.1. The Pre-Microfiltered Broth

The broth was obtained through the fermentation of KW. KW was collected from the HongboYuan dining hall, located in the University of Science and Technology Beijing, China. The collected KW was manually homogenized by first sorting out the coarse impurities, such as the hard bones, plastic bags, paper towels and other impurities, and then placed into the meat grinder for mixing well. Then, the KW serum was transferred into the self-sealing bag, and stored at −20 °C freezer [29]. After thawing, tap water was added to the KW serum with the ratio of 1:1 (*w*/*v*) ratio to adjust the total solids concentration. The LA bacteria adopted were *Montessori Enterococcus* CGMCC 22227, with the inoculation ratio of 10%, the fermentation temperature of 43 °C and the pH of 6.8–7.0 controlled by NaOH solution (10 mol/L) every 12 h.

After the 84 h of fermentation, the fermentation remnant was centrifuged by centrifuge (Hunan Hexi Instrument Equipment Co., Ltd., Changsha, China) (speed 12,000 rpm, centrifugation 10 min). After centrifugation, the bottom sedimentary substance and the upper oily substance were abandoned to obtain the raw broth. The raw fermented broth had a pH of 6.2–6.3, an LA content of 63.96 g/L, a chroma of 1137 Hazen, a turbidity of 175 NTU, a protein content of 0.21 g/L and a total sugar content of 3.33 g/L. Finally, the raw fermented broth was pre-treated by the MF process under the condition of the optimum pressure of 100 KPa, the pH of 6.0, and the backflushing mode with the deionized water for 3 min, and the obtained MF filtrate was the pre-microfiltered broth in this research with an LA content of 58.78 g/L, a chroma of 445.7 Hazen, a turbidity of 9.8 NTU, a protein content of 0.08 g/L and a total sugar content of 2.14 g/L.

#### 2.1.2. The Simulative Broth

To ensure the stability of the properties of the broth for comparison and selection of UF membranes, this study was equipped with simulative broth with reference to the previous research of our group. The simulative broth was prepared as follows: LA (47.9 g/L), bovine serum protein (2.5 g/L), glucose 3 g/L. 

### 2.2. Experimental Apparatus

**Figure 1:**
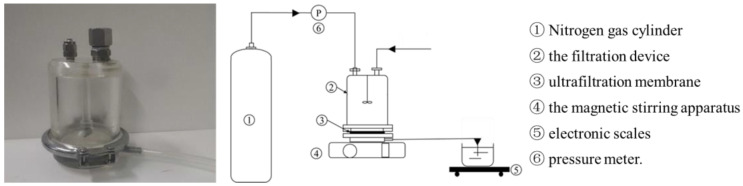
The illustration of the filtration configuration.

The UF apparatus adopted in this experiment was in a filtration cup configuration with a removable flat membrane structure (Shandong Bona Biotechnology Co., Ltd., Jinan, China), and the apparatus is shown in Figure 1. The magnetic stirring component was equipped to mix the liquid when conducting the filtration, and the nitrogen gas from the nitrogen gas cylinder (Beijing Huanyu Jinghui Gas Co., Ltd., Beijing, China) was used to force the fermented broth to pass through the membrane. The hydrophobic effect between the membrane surface and the proteins was deemed to be the dominant factor causing membrane fouling by the adsorption of proteins on the membrane. Thus, more hydrophilic membranes are usually favored [27]. In preliminary experiment, the MWCO range of under 150 KDa was proved to be reasonable for obtaining the desirable impurities, and so, based on the accessibility and cost, the 30 KDa, 50 and 100 KDa were selected. The four hydrophilic UF membranes were regenerated cellulose (RC) membranes (Shandong Bona Biotechnology Co., Ltd.) with a molecular weight cut off (MWCO) of 30 KDa (RC-30), 50 KDa (RC-50) and 100 KDa (RC-100), respectively, and polyethersulfone (PES) (Shandong Bona Biotechnology Co., Ltd.) membranes with a molecular weight of 50 KDa (PES-50) (In preliminary experiment, the 30 KDa PES and 100 KDa were also investigated. However, for the 30 KDa PES, the membrane flux was very low, and the blocking was serious. For the 100 KDa PES, the impurities removal efficiency was too poor. Thus, in this experiment, the 30 KDa PES and 100 KDa were excluded).

During the experiment, the UF membrane was set in the filtration cup, and then, a certain amount of fermented broth was loaded into the filtration cup, and the magnetic stirrer below was turned on at the speed of 600 rpm. Then, the nitrogen cylinder connected to the filtration cup was opened. By adjusting the pressure, the filtration was performed until the outflow velocity of the liquid was less than 0.2 mL/min.

When the UF membrane was not in use, it was stored in a container filled with desiccant. After use, it was kept in a wet state in 0.5% formaldehyde solution. It was soaked in deionized water for 1 h before each use.

### 2.3. Analysis Items and Methods

#### 2.3.1. Turbidity and Chroma

The turbidity and chroma of the broth were analyzed by the water quality analyzer (Beijing Lianhua Yongxing Science and Technology Development Co., Ltd., Beijing, China). The TRE and the CRE are calculated, as shown in Equations (1) and (2) [28].

(1) $$TRE=\frac{C_{n0}-C_{n}}{C_{n0}}\times 100$$

where C_n_ is the turbidity of the filtered transmissible fluid, NTU; C_n0_ is the turbidity of the fermentation broth before filtration, NTU.

(2) $$CRE=\frac{C_{h0}-C_{h}}{C_{h0}}\times 100$$

where C_h_ is the chromaticity of the filtered liquid, Hazen; C_h0_ is the chromaticity of the fermentation broth before filtration, Hazen.

#### 2.3.2. The Mass of LA

The determination of LA in broths was performed by high performance liquid chromatography (Shimadzu Corporation, Kyoto, Japan). 

The samples were centrifuged in a 12,000 r/min for 10 min, and the supernatant was filtered by a 0.22 μm membrane before determination. The shodex Sugar SH1011 liquid chromatography columns (8.0 mm × 300 mm) and the RID detector were adopted. Chromatographic conditions were column temperature: 60 °C; mobile Phase: 5 mM H2SO4; flow rate: 1.0 mL/min; injection volume: 10 μL. 

The calculation formula for the LRE is shown in Equation (3) [28].

(3) $$LRE=\frac{C_{L}^{\prime }V^{\prime }}{C_{L0}V_{0}}\times 100$$

where $C_{L}^{\prime }$ is the LA concentration of the fermented broth after filtration, g/L; V′ is the product of fermented liquid after filtration, L; C_L0_ is the LA concentration of fermentation broth before filtration, g/L; V_0_ is the product of fermentation liquid before filtration, L. 

#### 2.3.3. The Mass of Proteins

Proteins were determined using the Coomassie Brilliant Blue G-250 method. The PRE is calculated, as shown in Equation (4) [28].

(4) $$PRE=\frac{C_{p0}-C_{p}}{C_{p0}}\times 100$$

where C_p_ is the protein concentration in the filtered solution, g/L; C_p0_ is the protein concentration in fermentation broth before filtration, g/L.

#### 2.3.4. The Mass of Total Sugars

The determination of total sugars in the fermentation broth was carried out by the phenol-sulfuric acid method. The SRE is calculated, as shown in Equation (5) [28].

(5) $$SRE=\frac{C_{s0}-C_{s}}{C_{s0}}\times 100$$

where C_s_ is the total sugar concentration in the filtered permeable solution, g/L; C_s0_ is the total sugar concentration of fermentation broth before filtration, g/L.

#### 2.3.5. Membrane Flux

Membrane flux was defined as the volume of liquid that permeates a membrane per unit area per time. The flux calculation formula is shown in Equation (6) [28].

(6) $$Flux\;\left(\mathrm{mL}/\min\times m^{2}\right)=\frac{V}{t\times A}\times 100$$

where V is the filtrate, mL; t is the filtration time, min; A is the membrane area, m^2^.

#### 2.3.6. The Membrane Cleaning Performance 

The membrane cleaning performance was expressed by the membrane pure water flux recovery efficiency (FRE), which was calculated based on Equation (7) [28]

(7) $$FRE=\frac{J_{w\;}-J_{fw}}{J_{0\;}-J_{fw}}\times 100$$

where J_0_ is pure water permeation flux before membrane use, L/m^2^/h; J_fw_ is pure water permeation flux after membrane contamination, L/m^2^/h; J_w_ is pure water permeation flux after membrane cleaning, L/m^2^/h.

## 3. Results

### 3.1. Comparison and Selection of UF Membranes

At the room temperature of about 25 °C, and under the operating pressure of 120 KPa, four kinds of UF membranes were used to treat the simulative broth separately. The SRE, PRE and LRE were compared to select the suitable UF membrane for the refinement of LA from the pre-microfiltered broth.

**Figure 2:**
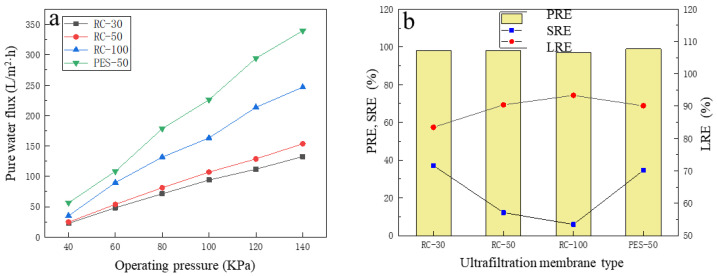
The selection of ultrafiltration membrane type: (**a**) the pure water flux of four kinds of ultrafiltration membrane under different operating pressure; (**b**) the filtration performance of four kinds of ultrafiltration membrane for protein, total sugar and lactic acid in simulated broth.

Under different pressures, the pure water flux of the four UF membranes changed, as shown in Figure 2a, and with the increase in the operating pressure, the pure water flux of the four membranes showed a linear upward trend. Under the same pressure, the pure water flux of the PES-50 membrane was significantly higher than that of the other three kinds of UF membranes, which indicated that the permeability of the PES-50 membrane was higher, and the membrane resistance was lower. It was explained by the hydrophobicity of RC membrane exceeding that of the PES membrane with the increase in pressure, as the higher the pressure, the more hydrophobic the membrane became [30]. When the simulative broth was filtered with four kinds of UF membranes, the performance of each membrane was shown in Figure 2b. The PREs were between 97.6% and 98.9%, which showed that the UF membrane had a very good removal performance for macromolecular proteins. Meanwhile, for the RC membrane, the PRE increased with the decrease in pore size, but the overall change range was very small, i.e., within 1%. Like protein, the SRE also increased with the decrease in pore size, but compared with the PRE, it was evident that the removal performance of UF membrane on total sugar was weak, i.e., between 5.9% and 37.0%. This was because the pore size determines the separation performance of UF: the UF process can effectively separate macromolecular substances (such as proteins), while most of the small molecular substances (such as glucose) will pass through the UF membrane [31,32]. For LA recovery, it was found that with the decrease in pore size, LRE showed a linear downward trend, and the decline was evident. For the PES-50 membrane, it was found that the SRE was 34.6% under the same MWCO, which was significantly higher than the RC-50 membrane (its SRE was only 12.2%). The RC-100 membrane had the lowest SRE, which indicated that the most sugar in the broth had lower MWCO. At the same time, the PES-50 membrane had the best PRE of 98.9%, while LRE of 90.1% was slightly lower than that of the RC-50 membrane (90.4%). Comprehensively, considering that the PES membrane had a high SRE and PRE, and the desirable LRE, PES-50 was finally selected for refining LA broth of KW fermentation.

### 3.2. The Effect of Operating Pressure on LA Refinement Performance

After the PES-50 membrane was selected, the actual pre-microfiltered broth was tested. To investigate the effect of operating pressure on LA refinement performance, experiments were carried out under different operating pressures. The change of membrane flux over the time was recorded, and the TRE, CRE, PRE, SRE and LRE were analyzed.

#### 3.2.1. The Change of UF Membrane Flux

**Figure 3:**
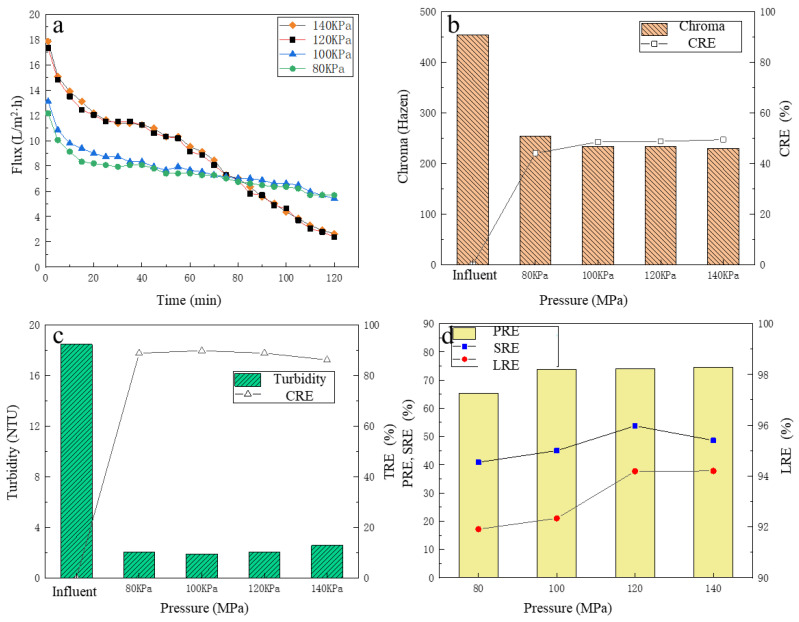
The effect of different pressure on filtration performance: (**a**) the flux change; (**b**) the chroma removal; (**c**) the turbidity removal; (**d**) the protein removal efficiency, the total sugar removal efficiency, and the lactic acid recovery efficiency.

As observed in Figure 3a, the higher the operating pressure, the higher the membrane flux. In initial time, the membrane flux decreased rapidly. For this phenomenon, the reason was inferred as at the beginning of filtration, the broth flowed to the surface of the membrane driven by the static pressure difference, and the macromolecular solute accumulated rapidly on the surface of the membrane due to the interception of the membrane. Thus, there existed concentration polarization phenomenon between the surface of the membrane and the broth, and the concentration polarization layer formed. The formation of concentration polarization made the resistance increase for broth reach the surface of the membrane. Thus, membrane flux decreased rapidly. With the filtration continued, the concentration polarization gradually reached a stable state, and the flux of the film maintained a stable state [33]. At 25–55 min, it was observed that the downward trend of membrane flux under four pressure conditions significantly reduced, and the membrane flux tended to be stable. When the operating pressure was below 120 KPa, the higher the operating pressure, the higher the membrane flux. However, when the operating pressure continued to increase to 140 KPa, the membrane flux basically did not increase, not even slightly less than the membrane flux under 120 KPa. This may have been due to the increased pressure caused concentration polarization and blockage of the membrane, then the formation of the cake layer caused a stable flux [34]. Additionally, the membrane flux under 120 KPa and 140 KPa was very close and significantly higher than that under 80 KPa and 100 KPa. After 55 min, the membrane fluxed under 120 KPa and 140 KPa, and began to plummet strictly again, while the membrane flux under 80 KPa and 100 KPa maintained a slow downward trend. It was probably because during the initial 55 min, with the high membrane flux under 120 KPa and 140 KPa, after most of the clear liquid having flowed out through the UF membrane, the concentrations of protein, total sugar and other substances in the remaining broth were significantly increased, which further aggravated the UF membrane pollution, and so, the membrane flux decreased rapidly [35]. Therefore, from the perspective of membrane flux, it was more appropriate to choose an operating pressure of 120 KPa.

#### 3.2.2. The Removal of Chroma and Turbidity

Before and after filtration, the chroma changes of the pre-microfiltered broth are shown in Figure 3b. The chroma of the filtrate was between 230–250 Hazen. It was clear that the CRE increased with the increase in operating pressure, reaching a maximum value of 49.38% under 140 KPa. Overall, the CRE fluctuated slightly. The reason may be that some macromolecular pigments were removed by the UF membrane, but the remaining pigment molecules with a relatively lower molecular weight [36] passed through the UF membrane, so that the chroma of the filtrate was still relatively high.

Before and after filtration, the turbidity of the pre-microfiltered broth changed, as shown in Figure 3c. As stated in Section 2.1.1, most of the insoluble particulate matter in the raw fermentation broth was removed by the pre-MF process, and only a small part of the particles with smaller particle size remained in the pre-microfiltered broth, which was further removed by UF membrane. The turbidity of the filtrate was basically between 2.1 and 2.8 NTU, which was extensively lower than that of the feed. With the operating pressure increased, the TRE of the fermentation broth increased first and then decreased, but the overall change was small. Under 120 KPa, the turbidity of the filtrate was as low as 2.14 NTU with the TRE of 85.62%.

#### 3.2.3. The Removal of Protein, Total Sugar and the LA Recovery

Under different operating pressures, the refinement performance of UF membrane for the pre-microfiltered broth is shown in Figure 3d. The PRE increased with the increase in the operating pressure. However, when the operating pressure was higher than 80 KPa, the increase in PRE was not obvious, i.e., around 74%. With the increase in operating pressure, the SRE was basically on the rise, reaching a maximum of 53.77% under 120 KPa. When the operating pressure further increased to 140 KPa, the SRE reduced to a certain extent, which was 48.62%. For LA recovery, up to 120 KPa, the LRE increased with the increase in the operating pressure. Under 120 KPa, the LRE was 94.19%. It was inferred that the higher the operating pressure, the larger the filtrate and the higher the LRE. When the operating pressure further increased to 140 KPa, the LRE remained basically unchanged. This was because when the pressure continued to increase, the membrane flux no longer increased, and the membrane surface contamination layer was tighter, finally hindering the passage of LA, resulting in an insignificant change in LA recovery. Considering the PRE, SRE and LRE, 120 KPa was finally selected as the optimal operating pressure for UF treatment of pre-microfiltered broth.

### 3.3. The Effect of pH on UF Performance

In addition to the operating pressure, the pH of the pre-microfiltered broth also had an impact on the UF process. To further investigate the effect of pH on UF performance, an HCl solution of 6 mol/L and a NaOH solution of 1 mol/L were used in this study to adjust the pH of the pre-microfiltered fermented broth to 5, 6, 7 and 8, respectively. UF experiments were performed under the operating pressure of 120 KPa, and the change of membrane flux over time was recorded, and the CRE, TRE, PRE and SRE at different pH were analyzed with the LRE. 

#### 3.3.1. The Change of Membrane Flux

**Figure 4:**
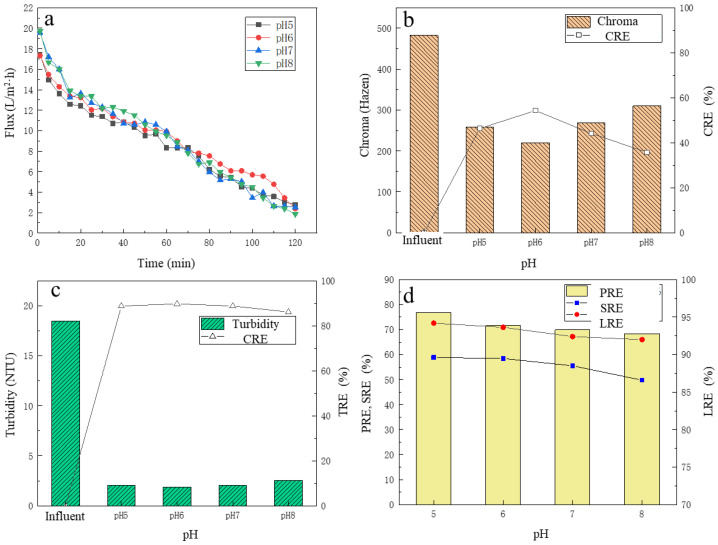
The effect of different pH on filtration performance: (**a**) the flux change; (**b**) the chroma removal; (**c**) the turbidity removal; (**d**) the protein removal efficiency, the total sugar removal efficiency and the lactic acid recovery efficiency.

From Figure 4a, when the pH of pre-microfiltered broth was fixed, the membrane flux gradually decreased over time. In the first 50 min, the average membrane fluxes at pH 5–8 were 12.3 L/(m^2^∙h), 12.8 L/(m^2^∙h), 13. 49 L/(m^2^∙h), 13.77 L/(m^2^∙h), respectively. Membrane flux increased with the increase in pH. This may have been partly because with the increase in pH, the repulsion between the functional groups of the membrane increased, resulting in a certain expansion of the membrane pores, resulting in a larger membrane flux. It was reported that with the increase in the pH, the membranes show anionic characteristics [37]. Thus, due to membranes and impurities mostly carrying a negative charge, electrostatic repulsion is helpful for decreasing fouling [38,39]. After 50 min, it was found that the membrane flux at pH 7 and pH 8 condition began to gradually decrease, which was lower than that under other pH conditions. The reason may have been because when pH increased, some of the substances in the pre-microfiltered broth, such as proteins, formed aggregates due to attractions of calcium complexes with phosphates and/or lactate and were deposited on the membrane surface, thereby reducing the membrane flux [40]. However, in general, although the membrane flux at different pH conditions varied, the gap was not obvious.

#### 3.3.2. The Removal of Chroma and Turbidity

Before and after filtration, the chroma changes of the pre-microfiltered broth are shown in Figure 4b. With the increase in pH, the chroma of the filtrate showed an upward trend, and the overall CRE showed a downward trend. At pH 6.0, the chroma of the filtrate was the lowest, i.e., only 220.2 Hazen, and the CRE reached 54.33%. Under different pH conditions, the turbidity changes are shown in Figure 4c. The turbidity of the pre-microfiltered broth was about 20 NTU, and the turbidity of the filtrate was basically 2.5 NTU or less. With the increase in pH of the pre-microfiltered broth, the turbidity of the filtrate showed an overall upward trend, and the TRE showed a downward trend. At pH 6.0, the turbidity of the filtrate was the lowest, only 1.89 NTU, and the TRE was 89.76%.

#### 3.3.3. The Removal of Protein, Total Sugar and the LA Recovery

Under different pH conditions, the refinement performance of UF membrane for the pre-microfiltered broth is shown in Figure 4d. With the increase in pH, PRE and SRE showed an overall downward trend, but the decline was relatively small. At pH 5, the PRE and SRE were the highest with the values of 76.84% and 58.86%, respectively, which indicated that acidic conditions facilitated the removal of protein and total sugar. With the increase in the pH, the LRE showed a downward trend. The LRE was 94.20% at pH 5.0 and was 92.00% at pH 8.0. This may be because as the pH increased, most of the LA was presented with the form of ions, which were easily repelled by negatively charged PES membranes, thereby increasing the LA loss [41]. By comprehensively considering TRE, CRE, PRE, SRE and LRE, it was found that lower pH conditions were more favorable for the UF performance. While considering that under low pH condition, the UF membrane flux would also decrease to a certain extent, and for achieving low the pH, the cost of hydrochloric acid regulation would increase, and so, the final choice was determined at pH 6.0 as the optimal pH condition for UF treatment.

### 3.4. The Effect of Cleaning Method on Polluted Membrane Recovery

#### 3.4.1. The Influence of Cleaning Mode on Cleaning Performance

**Figure 5:**
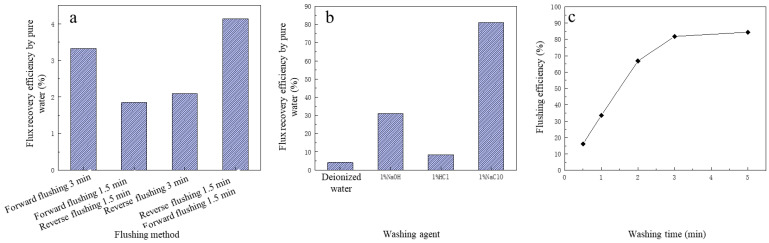
The effect of different flushing operations on flux recovery efficiency of microfiltration: (**a**) the flushing methods; (**b**) the flushing agents; (**c**) the effect of washing time on flushing efficiency.

In this study, deionized water was first used to clean the contaminated membrane, and the cleaning performance of four cleaning methods on the contaminated membrane was investigated. The four cleaning methods were as follows: (i) forward pressurized cleaning for 3 min, (ii) reverse pressurized cleaning for 3 min, (iii) forward pressurized cleaning for 1.5 min + reverse pressurized cleaning for 1.5 min and (iv) reverse pressurized cleaning for 1.5 min + forward pressurized washing 1.5 min. The pressure used for cleaning was 100 KPa. The cleaning performance of the four cleaning methods on contaminated UF membrane are shown in Figure 5.

From Figure 5a, it was obvious that with the same cleaning time, forward pressurized cleaning had a better effect than that of reverse pressurized cleaning to relieve membrane pollution, and the cleaning effect of forward pressurized cleaning + reverse pressurized cleaning was the worst, slightly lower than that of reverse pressurized cleaning. The reverse pressurized cleaning + forward pressurized cleaning had the best cleaning performance with the FRE of 4.13%. However, in general, regardless of which kind of cleaning method was adopted, the relief of membrane pollution was not desirable, and the maximum FRE was only 4.13%. Therefore, additional cleaning agents should be considered for enhanced cleaning effect.

#### 3.4.2. The Influence of Cleaning Agent on Cleaning Performance

Four cleaning agents were selected to further clean the contaminated membrane. It was reported that the concentration of cleaning chemicals is important to maintain the optimum reaction rate and overcome the mass transfer resistance of fouling layers [42]. The four cleaning agents were deionized water, 1% HCl, 1% NaOH and 1% NaClO; the cleaning method was first deionized water forward pressure cleaning for 3 min, then cleaning agent reverse pressurized for 3 min with the cleaning pressure of 100 KPa. The performance of four kinds of cleaning agents on UF membrane pollution are shown in Figure 5b. The FRE with deionized water was the worst, only 4.19%, which was 50% of that with HCl. The cleaning performance of NaOH was obviously better than that with HCl, and the FRE was 31.17%. NaClO had the best cleaning effect with FRE reaching 81.19%, which was much higher than the other three cleaning agents. The reason may be that as membrane pollution was mainly caused by organic matter, the NaClO pair had strong oxidation, which could oxidize and remove most organic pollutants [43]. Therefore, 1% NaClO was finally selected as the cleaning agent. In addition, it should be mentioned that while chemical cleaning is an effective operation, it may be harsh to the membrane and damage the membrane material, resulting in reduced membrane lifetime and selectivity [44].

#### 3.4.3. The Influence of Cleaning Time on Cleaning Performance

After the cleaning agent was selected with 1% NaClO, to further improve the cleaning efficiency, this section investigated the effect of cleaning time on cleaning effect. As shown in Figure 5c, within the first 3 min of cleaning, the FRE increased significantly with the increase in cleaning time, and when the cleaning time was 30 s, the FRE was only 16.22%, and when the cleaning time reached 3 min, the FRE increased to 81.19%. However, when the cleaning time further increased to 5 min, the FRE was 84.43%, which was slightly higher than that at 3 min. It was also reported that FRE increased by increasing the cleaning time. However, the FRE slowly decreased due to the limited chemicals to dissolve the deposited layer [45]. The other study also showed that FRE did not depend on the cleaning time, and short intensive cleaning was better than long comprehensive cleaning [46]. Therefore, to save cleaning time and the amount of cleaning agent, the more reasonable cleaning time in NaClO cleaning method was determined to be 3 min. 

#### 3.4.4. Membrane Surface Morphology Analysis

**Figure 6:**
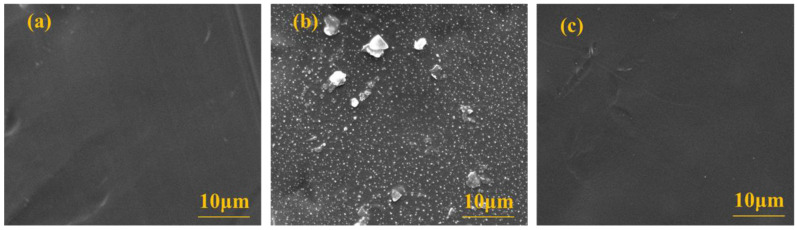
The surface observation of the microfiltration membrane by SEM instrument: (**a**) the new membrane; (**b**) after the blocking; (**c**) after the flushing with 1% NaClO.

As shown in Figure 6, the surface morphology changes of the UF membrane observed through scanning electron microscopy were determined. The surface of the new UF membrane was smooth and basically free of particulate matter. There were obviously much particulate contaminants on the surface of the contaminated UF membrane, and most of the pollutants had a small particle size of about 0.5 μm. After cleaning with 1% NaClO solution, the surface pollution of the membrane was basically relieved, which fully indicated that NaClO had a significant removal effect on the surface of the UF membrane. No matter the dead-end or the crossflow UF, the membrane fouling was related to the feed feature, such as the impurity’s concentration, and the filtration time. In this research, although the dead-end UF was adopted, the magnetic stirring component was equipped to relief the fouling. The increase in the concentration of solution was reflected in the membrane performance, which was same to the feed with high concentration. 

### 3.5. The Effect of Cleaning Cycle on UF Membrane Performance

**Figure 7:**
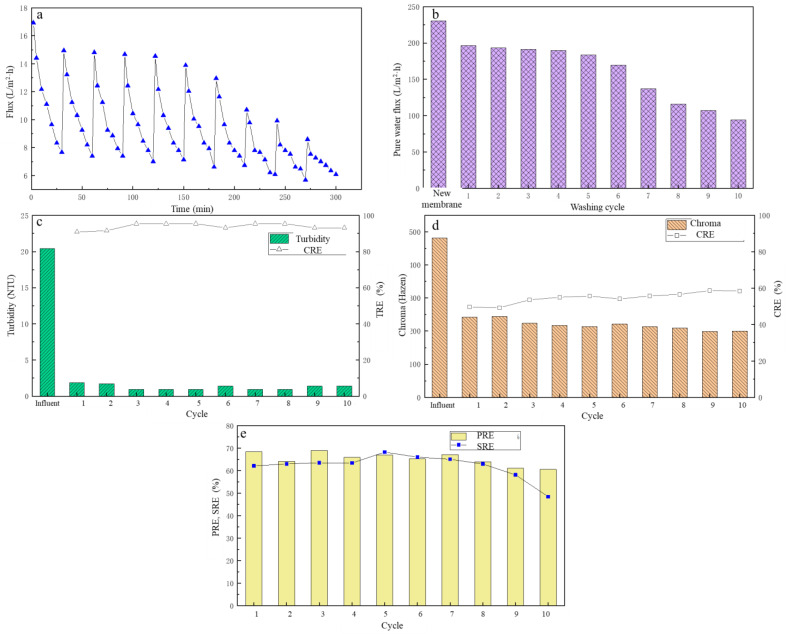
The effect of different flushing cycles on filtration performance: (**a**) the flux change; (**b**) the pure water flux; (**c**) the turbidity removal; (**d**) the chroma removal; (**e**) the protein removal efficiency and the total sugar removal efficiency.

After the cleaning agent and cleaning method were determined, the 10 cycles of UF experiment were carried out. Each cycle ran for 30 min and the change of membrane flux over time was recorded. After the filtration was completed, the UF membrane was rinsed with 1% NaClO. The pure water fluxes of the UF membrane in each cycle were recorded, the treatment performance of each filtration cycle was analyzed, as shown in Figure 7a–e.

From Figure 7a, the membrane flux decreased with the increase in the filtration cycle. However, from circle two to six, the membrane flux decreased significantly. The average membrane flux was maintained at around 10.1 L/(m^2^∙h), the effectiveness of the cleaning effect of NaClO was fully demonstrated. However, from the seventh cycle, the membrane flux began to decline sharply, and the trend of the pure water flux of the membrane was also the same, as shown in Figure 7b. When the cycles of membrane cleanings were less than six, the pure water flux was basically maintained at 180–190 L/(m^2^∙h). However, when the cycles of cleanings were more than six, the pure water flux of the membrane began to decline rapidly. After 10 cycles, the membrane had a pure water flux of only 94 L/(m^2^∙h), which was 41% of the pure water flux of the new membrane. The reason for this may be that NaClO caused some damage to the membrane pores, resulting in a decrease in membrane flux [44]. 

Under different filtration cycles, the TRE and CRE are shown in Figure 7c,d. After filtration, the turbidity of the filtrate was basically below 2 NTU. Although the TRE fluctuated, it was basically between 90% and 95%, and the variation was small. The CRE showed an overall upward trend with the increase of the cycles, basically maintaining between 50 and 60%, and in the ninth cycle, the CRE reached the highest value of 58.61%.

Under different filtration cycles, the PREs and SREs from the fermentation broth are shown in Figure 7e. The PREs were basically between 64% and 69% in the first eight cycles. From the eighth cycle, the PRE began to gradually decrease. The SRE increased first and then decreased with the increase in the cycle, reaching the highest in the fifth cycle, which was 68.24%. Overall, the change was small in the first eight cycles with the value of about 64%. In the 10th cycle, it had the lowest PRE and SRE of 60.57% and 48.4%, respectively. After comprehensive analysis, after six cycles, the membrane could still maintain a good membrane flux and treatment performance. 

### 3.6. The Whole Evaluation of the UF

**Figure 8:**
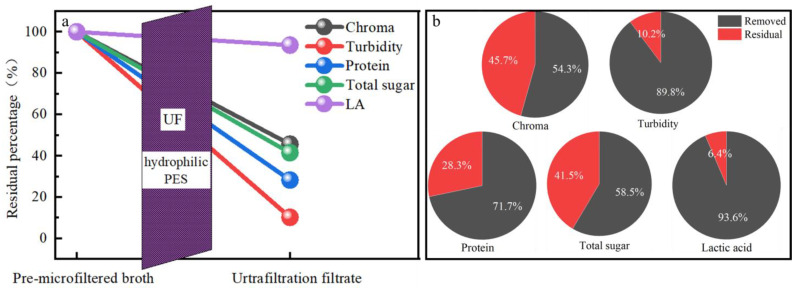
The whole removal performance of the microfiltration membrane separation (**a**) and the removal percentage of each component (**b**).

The whole removal performance of the UF process in this study was summarized, as shown in Figure 8. For all the impurities, UF process achieved the best removal performance with TRE of above 89.8%, followed by the CRE of 54.3%, PRE of above 71.7%, and SRE of 58.5%. The final UF filtrate had the LA content of 55.0 g/L, the chroma of 203.7 Hazen, the turbidity of 1.0 NTU, the protein content of 0.02 g/L and the total sugar content of 0.9 g/L. From these, it was obvious that for the refinement of the LA from the pre-microfiltered broth, the UF process could effectively achieve the target to some extent. The chroma was still high, which indicated that the combination of UF with other post-treatment processes is needed. In addition, the LA loss was about 6.4%. Thus, in future research, the effort to further lessen the loss of LA during the filtration should also be mentioned. 

## 4. Conclusions

In this study, for the LA refinement from pre-microfiltered fermentation broth, with the 50 KDa polyethersulfone membrane, the optimum operation parameters were determined as the pressure of 120 KPa, the pH of 6.0 and the flushing mode of backwashing with 1% NaClO for 3 min. The best performance was achieved with the CRE, TRE, PRE and SRE of 54.3%, 89.8%, 71.7% and 58.5%, respectively, and LRE was 93.6%. This study paves the way to LA refinement with UF membrane technology. The obtained results had the meaning as the reference for the future research. In conclusion, the UF process is promising as the effective separation method for the preliminary refinement of fermentation broth through combing with other pre-treatment processes and post-treatment processes.

## Data Availability

A statement was submitted.

## References

[1] Huang Y.L., Wu Z., Zhang L., Ming Cheung C., Yang S.-T. (2002). Production of Carboxylic Acids from Hydrolyzed Corn Meal by Immobilized Cell Fermentation in a Fibrous-Bed Bioreactor. Bioresour. Technol..

[2] Lee Y., Lee J.H., Yang H.J., Jang M., Kim J.R., Byun E.-H., Lee J., Na J.-G., Kim S.W., Park C. (2017). Efficient Simultaneous Production of Biodiesel and Glycerol Carbonate via Statistical Optimization. J. Ind. Eng. Chem..

[3] Berhanu M., Jabasingh S.A., Kifile Z. (2017). Expanding Sustenance in Ethiopia Based on Renewable Energy Resources—A Comprehensive Review. Renew. Sustain. Energy Rev..

[4] Ma X., Gao M., Liu S., Li Y., Sun X., Wang Q. (2022). An Innovative Approach for Reducing the Water and Alkali Consumption in the Lactic Acid Fermentation via the Reuse of Pretreated Liquid. Bioresour. Technol..

[5] Xu M., Sun H., Yang M., Xie D., Sun X., Meng J., Wang Q., Wu C. (2022). Biodrying of Biogas Residue through a Thermophilic Bacterial Agent Inoculation: Insights into Dewatering Contribution and Microbial Mechanism. Bioresour. Technol..

[6] Zacharof M.-P., Lovitt R.W. (2013). Recovery of Volatile Fatty Acids (VFA) from Complex Waste Effluents Using Membranes. Water Sci. Technol..

[7] Aghapour Aktij S., Zirehpour A., Mollahosseini A., Taherzadeh M.J., Tiraferri A., Rahimpour A. (2020). Feasibility of Membrane Processes for the Recovery and Purification of Bio-Based Volatile Fatty Acids: A Comprehensive Review. J. Ind. Eng. Chem..

[8] Raposo F., Borja R., Cacho J.A., Mumme J., Orupõld K., Esteves S., Noguerol-Arias J., Picard S., Nielfa A., Scherer P. (2013). First International Comparative Study of Volatile Fatty Acids in Aqueous Samples by Chromatographic Techniques: Evaluating Sources of Error. TrAC Trends Anal. Chem..

[9] Fernández R., Dinsdale R.M., Guwy A.J., Premier G.C. (2016). Critical Analysis of Methods for the Measurement of Volatile Fatty Acids. Crit. Rev. Environ. Sci. Technol..

[10] Masse L., Massé D.I., Pellerin Y. (2008). The Effect of PH on the Separation of Manure Nutrients with Reverse Osmosis Membranes. J. Memb. Sci..

[11] Huang C., Xu T., Zhang Y., Xue Y., Chen G. (2007). Application of Electrodialysis to the Production of Organic Acids: State-of-the-Art and Recent Developments. J. Memb. Sci..

[12] de-Bashan L.E., Bashan Y. (2004). Recent Advances in Removing Phosphorus from Wastewater and Its Future Use as Fertilizer (1997–2003). Water Res..

[13] Gluszcz P., Jamroz T., Sencio B., Ledakowicz S. (2004). Equilibrium and Dynamic Investigations of Organic Acids Adsorption onto Ion-Exchange Resins. Bioprocess. Biosyst. Eng..

[14] Senol A., Dramur U. (2004). Predicting Liquid–Liquid Equilibria of Amine Extraction of Carboxylic Acid Through Solvation Energy Relation. Solvent Extr. Ion Exch..

[15] Mumtaz T., Abd-Aziz S., Aini N., Rahman A., Yee P., Shirai Y., Hassan M. (2008). Pilot-Scale Recovery of Low Molecular Weight Organic Acids from Anaerobically Treated Palm Oil Mill Effluent (POME) with Energy Integrated System. Afr. J. Biotechnol..

[16] Lo K.-M., Chien I.-L. (2017). Efficient Separation Method for Tert-Butanol Dehydration via Extractive Distillation. J. Taiwan Inst. Chem. Eng..

[17] Joglekar H.G., Rahman I., Babu S., Kulkarni B.D., Joshi A. (2006). Comparative Assessment of Downstream Processing Options for Lactic. Acid. Sep. Purif. Technol..

[18] Manzak A., Kurşun C., Yıldız Y. (2017). Characterization of Humic Acid Extracted from Aqueous Solutions with Polymer Inclusion Membranes. J. Taiwan Inst. Chem. Eng..

[19] Trad Z., Akimbomi J., Vial C., Larroche C., Taherzadeh M.J., Fontaine J.-P. (2015). Development of a Submerged Anaerobic Membrane Bioreactor for Concurrent Extraction of Volatile Fatty Acids and Biohydrogen Production. Bioresour. Technol..

[20] Bastrzyk J., Gryta M., Karakulski K. (2014). Fouling of Nanofiltration Membranes Used for Separation of Fermented Glycerol Solutions. Chem. Pap..

[21] Bonnélye V., Guey L., del Castillo J. (2008). UF/MF as RO Pre-Treatment: The Real Benefit. Desalination.

[22] Karakulski K., Gryta M., Bastrzyk J. (2013). Treatment of Effluents from a Membrane Bioreactor by Nanofiltration Using Tubular Membranes. Chem. Pap..

[23] Tomczak W., Gryta M. (2013). The Application of Ultrafiltration for Separation of Glycerol Solution Fermented by Bacteria. Pol. J. Chem. Technol..

[24] Mao C., Feng Y., Wang X., Ren G. (2015). Review on Research Achievements of Biogas from Anaerobic Digestion. Renew. Sustain. Energy Rev..

[25] Wang Q., Li H., Feng K., Liu J. (2020). Oriented Fermentation of Food Waste towards High-Value Products: A Review. Energies.

[26] Dahiya S., Sarkar O., Swamy Y.v., Venkata Mohan S. (2015). Acidogenic Fermentation of Food Waste for Volatile Fatty Acid Production with Co-Generation of Biohydrogen. Bioresour. Technol..

[27] Gryta M., Tomczak W. (2015). Microfiltration of Post-Fermentation Broth with Backflushing Membrane Cleaning. Chem. Pap..

[28] Sun X., Wang Q., Zhao W., Ma H., Sakata K. (2006). Extraction and Purification of Lactic Acid from Fermentation Broth by Esterification and Hydrolysis Method. Sep. Purif. Technol..

[29] Gao Z., Ma Y., Liu Y., Wang Q. (2022). Waste Cooking Oil Used as Carbon Source for Microbial Lipid Production: Promoter or Inhibitor. Environ. Res..

[30] Puro L., Kallioinen M., Mänttäri M., Natarajan G., Cameron D., Nyström M. (2010). Performance of RC and PES Ultrafiltration Membranes in Filtration of Pulp Mill Process Waters. Desalination.

[31] Charcosset C. (2021). Classical and Recent Applications of Membrane Processes in the Food Industry. Food Eng. Rev..

[32] Castro-Muñoz R., Díaz-Montes E., Cassano A., Gontarek E. (2021). Membrane Separation Processes for the Extraction and Purification of Steviol Glycosides: An Overview. Crit. Rev. Food Sci. Nutr..

[33] Adikane H., Thakar D., Nene S. (2004). Optimisation of Colour and Sugar Rejection of Black Liquor Using Membranes. Sep. Purif. Technol..

[34] Sadr S.M.K., Saroj D.P., Basile A., Cassano A., Rastogi N.K. (2015). Membrane Technologies for Municipal Wastewater Treatment. Advances in Membrane Technologies for Water Treatment.

[35] Li S., Chen H., Zhao X., Lucia L.A., Liang C., Liu Y. (2020). Impact Factors for Flux Decline in Ultrafiltration of Lignocellulosic Hydrolysis Liquor. Sep. Purif Technol..

[36] Hidalgo A.M., Gómez M., Murcia M.D., Serrano M., Rodríguez-Schmidt R., Escudero P.A. (2018). Behaviour of Polysulfone Ultrafiltration Membrane for Dyes Removal. Water Sci. Technol..

[37] Díaz-Montes E., Castro-Muñoz R. (2019). Metabolites Recovery from Fermentation Broths via Pressure-Driven Membrane Processes. Asia-Pac. J. Chem. Eng..

[38] Huang S., Ras R.H.A., Tian X. (2018). Antifouling Membranes for Oily Wastewater Treatment: Interplay between Wetting and Membrane Fouling. Curr. Opin. Colloid Interface Sci..

[39] Madaeni S.S., Sharifnia S., Moradi G. (2001). Chemical Cleaning of Microfiltration Membranes Fouled by Whey. J. Chin. Chem. Soc..

[40] Chandrapala J., Duke M.C., Gray S.R., Weeks M., Palmer M., Vasiljevic T. (2017). Strategies for Maximizing Removal of Lactic Acid from Acid Whey—Addressing the Un-Processability Issue. Sep. Purif. Technol..

[41] Koschuh W., Thang V.H., Krasteva S., Novalin S., Kulbe K.D. (2005). Flux and Retention Behaviour of Nanofiltration and Fine Ultrafiltration Membranes in Filtrating Juice from a Green Biorefinery: A Membrane Screening. J. Memb. Sci..

[42] Paugam L., Delaunay D., Diagne N.W., Rabiller-Baudry M. (2013). Cleaning of Skim Milk PES Ultrafiltration Membrane: On the Real Effect of Nitric Acid Step. J. Memb. Sci..

[43] Hou L., Gao K., Li P., Zhang X., Wang Z., Song P., Yao W. (2017). A Kinetic Model for Calculating Total Membrane Fouling Resistance in Chemical Cleaning Process. Chem. Eng. Res. Des..

[44] Bird M.R., Bartlett M. (2002). Measuring and Modelling Flux Recovery during the Chemical Cleaning of MF Membranes for the Processing of Whey Protein Concentrate. J. Food Eng..

[45] Madaeni S., Tavakolian H., Rahimpour F. (2011). Cleaning Optimization of Microfiltration Membrane Employed for Milk Sterilization. Sep. Sci. Technol..

[46] Naim R., Levitsky I., Gitis V. (2012). Surfactant Cleaning of UF Membranes Fouled by Proteins. Sep. Purif. Technol..

